# Supplementary figures and images for: Density Gradient Centrifugation Is an Effective Tool to Isolate Cancer Stem-like Cells from Hypoxic and Normoxia Triple-Negative Breast Cancer Models
Source: Int J Mol Sci. 2024 Aug 17;25(16):8958. doi: 10.3390/ijms25168958 (PMC11354270; doi:10.3390/ijms25168958)

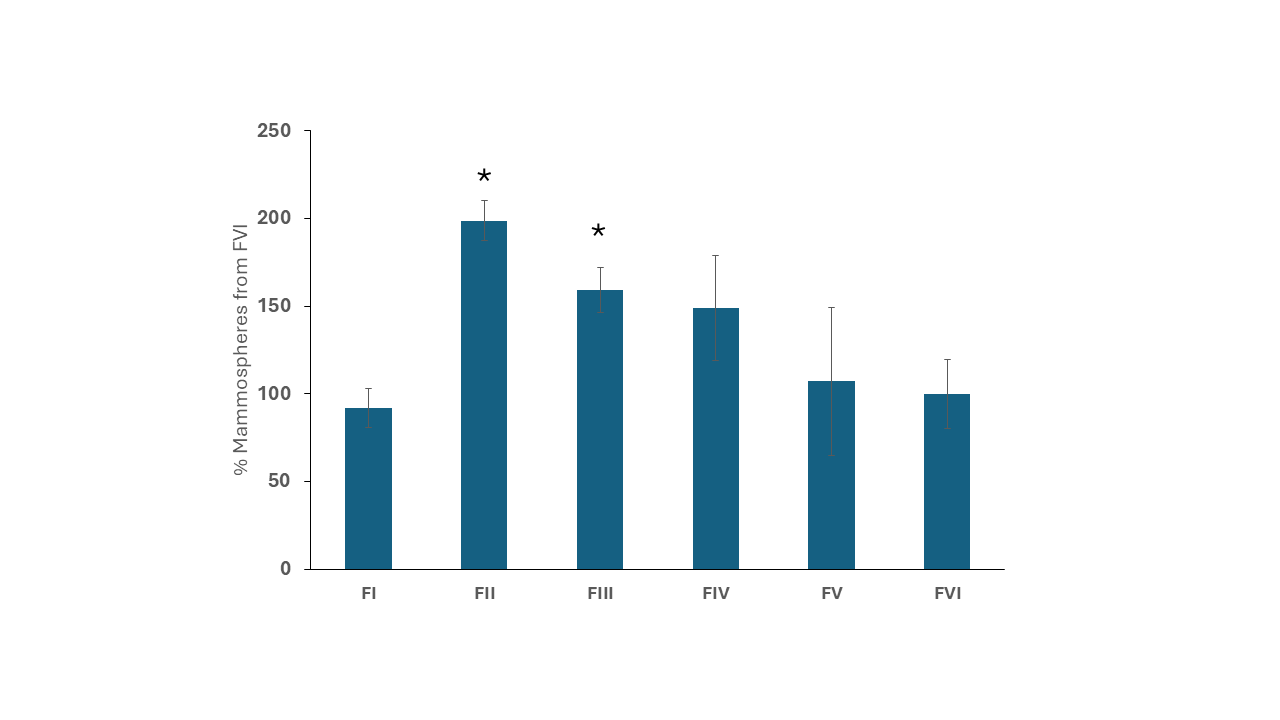

Supplement: Supplementary file 1 [file ijms-25-08958-s001.zip › Figure S1.tif]

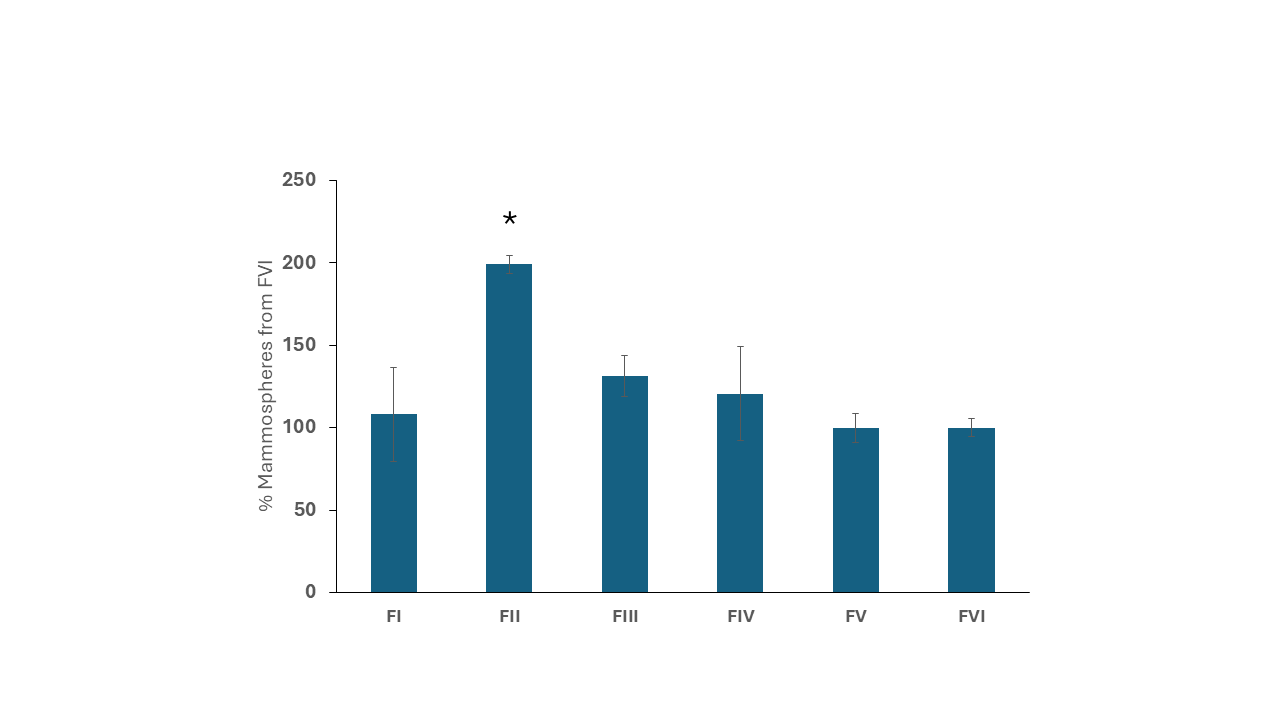

Supplement: Supplementary file 1 [file ijms-25-08958-s001.zip › Figure S2.tif]

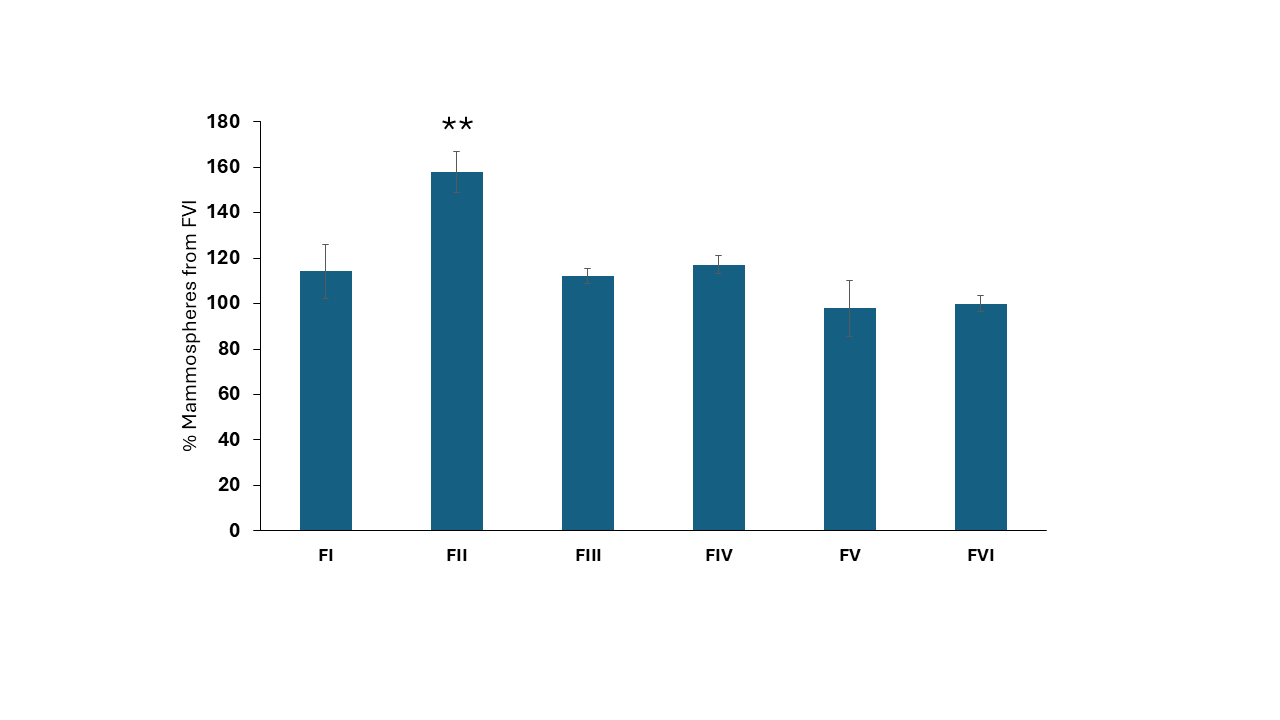

Supplement: Supplementary file 1 [file ijms-25-08958-s001.zip › Figure S3.tif]

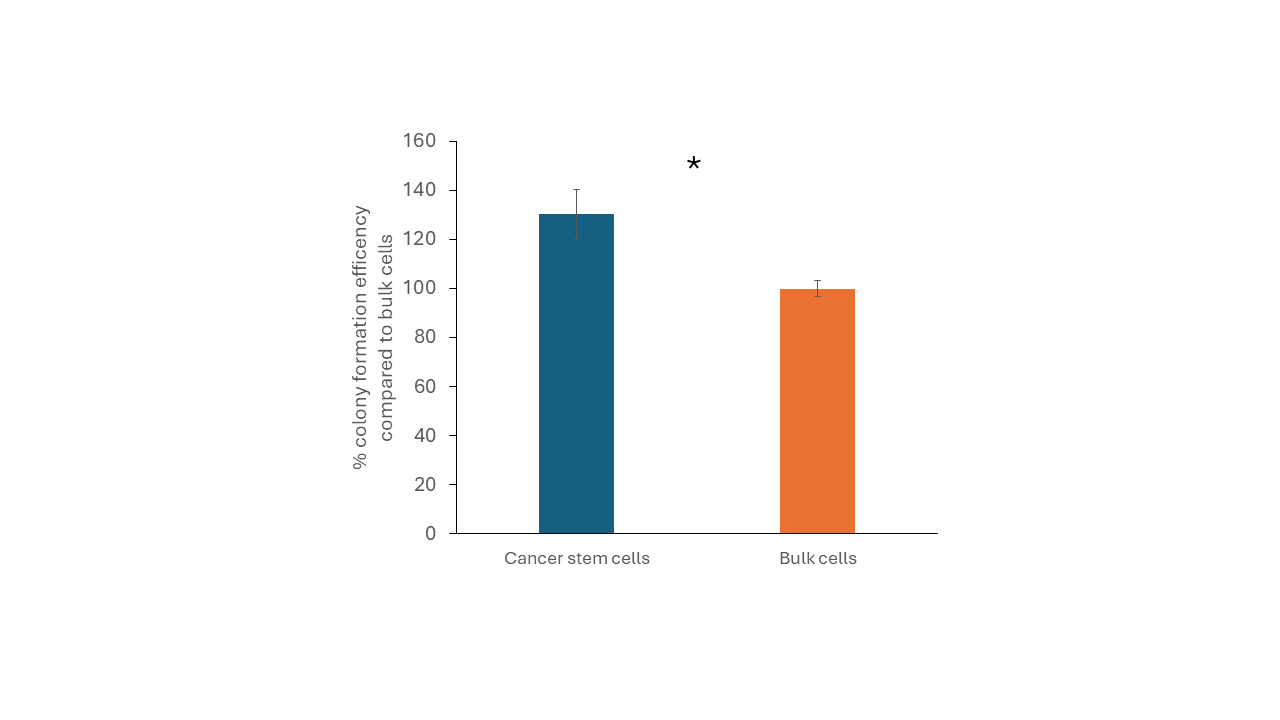

Supplement: Supplementary file 1 [file ijms-25-08958-s001.zip › Figure S4.tif]

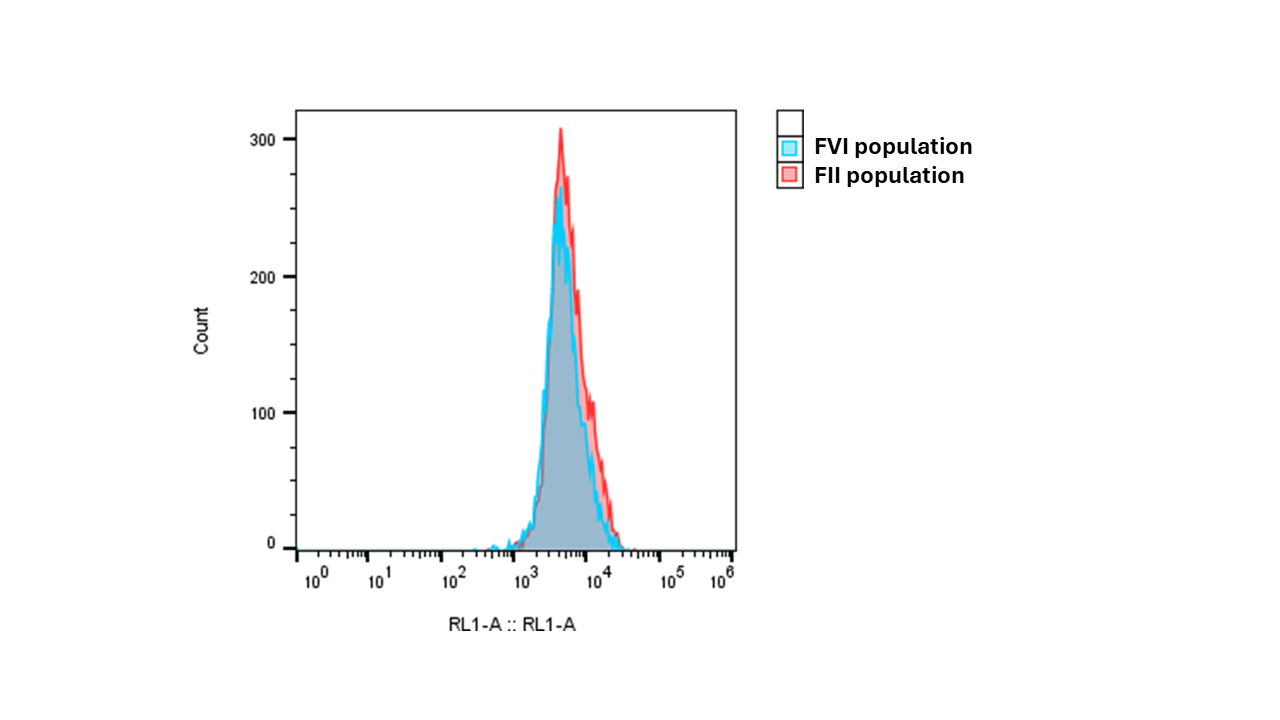

Supplement: Supplementary file 1 [file ijms-25-08958-s001.zip › Figure S5.TIF]

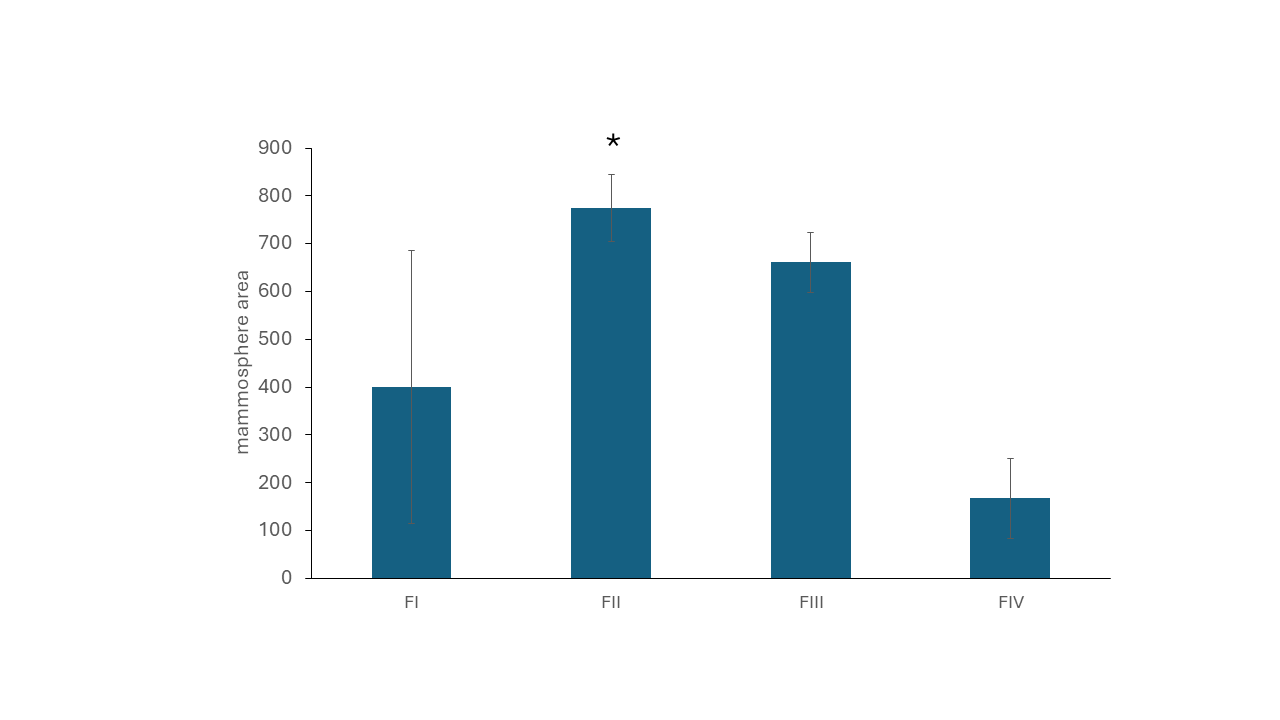

Supplement: Supplementary file 1 [file ijms-25-08958-s001.zip › Figure S6.tif]
